# A Molecular and Clinical Review of Stem Cell Therapy in Critical Limb Ischemia

**DOI:** 10.1155/2017/3750829

**Published:** 2017-11-20

**Authors:** Punam P. Parikh, Zhao-Jun Liu, Omaida C. Velazquez

**Affiliations:** DeWitt-Daughtry Family Department of Surgery, Division of Vascular Surgery, University of Miami Miller School of Medicine, Miami, FL, USA

## Abstract

Peripheral artery disease (PAD) is one of the major vascular complications in individuals suffering from diabetes and in the elderly that can progress to critical limb ischemia (CLI), portending significant burden in terms of patient morbidity and mortality. Over the last two decades, stem cell therapy (SCT) has risen as an attractive alternative to traditional surgical and/or endovascular revascularization to treat this disorder. The primary benefit of SCT is to induce therapeutic neovascularization and promote collateral vessel formation to increase blood flow in the ischemic limb and soft tissue. Existing evidence provides a solid rationale for ongoing in-depth studies aimed at advancing current SCT that may change the way PAD/CLI patients are treated.

## 1. Introduction

Peripheral artery disease (PAD) is a progressive atherosclerotic disorder that can lead to poor quality of life, high cost of care, and an increased risk of hospitalization and mortality [1–3]. As a consequence, the prevalence of PAD increases with age due to persistent rates of tobacco use, an increase in type 2 diabetes, obesity, and sedentary lifestyle [4]. From 2003 to 2012, the prevalence of PAD among those aged ≥ 75 years increased from 12.5% in 2003 to 18.5% in 2012, where the mean age-standardized incidence of PAD across all observation years was 26.8 per 1000 person-years [1].

As the most severe form of PAD, atherosclerosis-mediated critical limb ischemia (CLI) represents the main cause of ischemic rest pain, nonhealing ulcers, and gangrene or tissue loss. The estimated incidence of CLI is 160,000 in the United States, where 5–10% of patients with asymptomatic PAD will progress to CLI five years from initial diagnosis [1, 5]. Furthermore, diabetic patients are fivefold more likely to develop CLI as compared to nondiabetic patients and are up to 40 times more likely to necessitate lower limb amputation, manifesting an even higher rate of morbidity and mortality [1]. As a hallmark of florid PAD, CLI remains an important condition in the general population with a strong socioeconomic burden that necessitates patient-tailored treatment.

Ischemic injury in normal tissue is characterized by a revascularization compensatory response including angiogenesis and arteriogenesis, but this response is defective in CLI [6]. As a result, the treatment of CLI patients is multidisciplinary. Currently, the standard of therapy for improving blood flow to the affected extremity is either surgical or endovascular revascularization [7, 8]. However, approximately 20% to 40% of patients are unsuitable for such interventions due to high operative risk or unfavorable endovascular anatomy [9]. Underlying atherosclerosis may be treated pharmacologically using lipid reduction, antiplatelet, and antihypertensive therapies, none of which have been proven effective in reducing amputation rates in CLI patients [10, 11]. Furthermore, there are no Food and Drug Administration- (FDA-) approved therapies for CLI. Oftentimes, the last resort for these patients who have exhausted their surgical options is management of associated comorbidities with intensive wound care, pain control, and eventual limb amputation. It is estimated that the mortality rate in these patients who are not eligible for surgical revascularization or endovascular treatment within six months from diagnosis is approximately 20%, while another 40% undergo major limb amputation [12]. Considering the limitations of current therapies and high rate of mortality, CLI quality of life has ultimately been likened to that of terminal cancer [13].

The no-option CLI patient represents a population with a serious, life-threatening disease and an unmet medical need. Novel and more effective strategies including stem cell therapy (SCT) have emerged as a promising alternative for treatment of disorders related to limb ischemia [13–17]. The purpose of this review is to provide an overview of the molecular etiology of stem cells and highlight their clinical applicability as an alternative treatment modality in patients with PAD/CLI.

## 2. Background on Stem Cells

### 2.1. Derivation of Stem Cells

Biologic regenerative therapies, including SCT, are currently undergoing clinical investigation. In several clinical studies, administration of bone marrow (BM) cells appears to have improved CLI patient outcomes [13, 18–20]. Stem cells have the competency to self-renew indefinitely while maintaining the potential to differentiate and give rise to any mature cell type in the human body. The process of self-renewal entails either symmetric division, creating two identical daughter cells endowed with stem cell properties, or asymmetric division, forming one stem cell and one progenitor cell with limited self-renewal and early maturation. In mammals, there are two broad types of stem cells, embryonic stem cells (ESCs) and “somatic” or adult stem cells. ESCs are isolated from the inner cell mass of a blastocyst or an early-stage embryo. These totipotent stem cells not only differentiate into all specialized cells such as ectoderm, endoderm, and mesoderm but can also maintain the normal turnover of regenerative organs such as blood, skin, and intestinal tissue. Originally used for reproductive purposes through *in vitro* fertilization in the 1990s, human ESCs have since been donated for investigation of their promising role in regenerative medicine. However, human ESC research remains ethically and politically controversially given that it involves the destruction of human embryos.

Conversely, in adults, the BM is a reservoir for stem and progenitor cells that can repair damaged tissue through paracrine mechanism and/or differentiation into appropriate tissue cells. However, adult stem cells have limitations regarding their potency; unlike ESCs, they are not able to differentiate into cells from all three germ layers and are thereby deemed multipotent versus totipotent cells. However, reprogramming allows for the creation of induced pluripotent stem cells (iPS cells) from adult cells. These are not adult stem cells but rather adult mature cells that are reprogrammed to give rise to cells with pluripotent capabilities akin to ESCs. In 2006, the first demonstration of iPS cells was conducted where four specific protein transcription factors, Oct3/4, Sox2, c-Myc, and Klf4, were used to reprogram mouse fibroblast cells into pluripotent cells, a finding subsequently reproduced using human dermal fibroblast cells [21, 22]. Human iPS cells were found to be similar to ESCs in morphology, proliferation, gene expression, surface markers, telomerase activity, *in vitro* differentiation, and teratoma formation [22].

There are several known accessible sources of adult stem cells: (1) BM, which requires extraction by harvesting tissue from typically the femur or iliac crest, giving rise to BM-derived endothelial progenitor cells (EPCs), BM-derived mesenchymal stem cells (MSCs), and BM-derived mononuclear cells (MNCs); (2) adipose tissue (lipid cells), which requires extraction by liposuction; (3) blood, which requires extraction through apheresis; (4) umbilical cord blood, which is extracted just after birth; and (5) placenta [23–27]. Stem cells may be autologous, which carry the least risk for adverse reaction, as opposed to allogeneic stem cells, derived from young, healthy donors. Furthermore, several studies have shown that like BM-derived EPCs, BM-derived hematopoietic progenitors may also differentiate to restore tissue vascularization after ischemic events not only in limbs but also in the retina and myocardium [28–30].

### 2.2. Mobilization of Stem Cells

Pathophysiologically, CLI is the result of systemic atherosclerosis-induced macrovascular lesions to the lower extremity that reduce distal perfusion, producing severe alteration of nutrients and blood flow within the microcirculation [1]. Ultimately, chronic ischemia hinders tissue capacity for oxygen diffusion and nutrients from peri-ischemic territories, as well as for endogenous remodeling [1]. Recent therapeutic strategies have focused on restoring this balance in favor of tissue survival using SCT. Dysfunction in the vascular bed in ischemic conditions, attrition of the microvasculature, and the difficulty or impossibility to adapt to the need for increased blood flow are critical points through which we investigate selective recruitment of stem and progenitor cells to the ischemic limb.

Several studies in mice indicate that the BM is likely to be a central source for mobilized stem and progenitor cells [31–33]. Emigration of cells from the BM is generally thought to occur after a period of cell proliferation within the marrow niche [34]. In mice, infusion of soluble kit ligand triggers mobilization of CD34^+^ stem cells within one hour [35]. In humans, there is a fourfold elevation in circulating EPCs within ten minutes of highly strenuous exercise [36]. It appears that there is a subpopulation of BM-derived EPCs within a specialized BM niche that is poised for rapid release to the circulation [35]. The increased systemic release of BM-derived EPCs into circulation improves neovascularization and wound healing in murine ischemic excisional hindlimb wounds [31].

Nitric oxide (NO) has been shown to play a central role in BM mobilization and release of EPCs [37]. Using ischemic and diabetic murine models, hyperoxia, induced by a clinically relevant hyperbaric oxygen protocol, increases NO levels within femoral BM, accelerates the spontaneous revascularization of surgically induced hindlimb ischemia, and increases the number of BM-derived EPCs in circulation within cutaneous hindlimb ischemic and diabetic wounds [37]. The hyperbaric oxygen-mediated elevation in circulating CD34^+^ stem cells results in an increase in colony forming cell capacity of circulating cells, an effect that is inhibited by pretreatment with L-nitroarginine methyl ester (L-NAME), a NO synthase inhibitor [37]. Overall, these effects on BM-derived EPC mobilization, vasculogenesis, and wound-healing were not observed in mice that received treatment with L-NAME prior to hyperbaric oxygen, indicating that the aforementioned improvements are mediated by NO [37].

While hyperoxia therapeutically stimulates stem and progenitor cell release from the BM, these cells may be effectively recruited to wounds to enhance vasculogenesis and healing only if the cytokine milieu in the cutaneous wound bed is optimized [31, 32]. Cytokines such as granulocyte colony-stimulating factor (G-CSF) and growth factors such as vascular endothelial growth factor-A (VEGF-A) have been shown to enhance trafficking of hematopoietic stem cells from their BM niche to the peripheral bloodstream [37]. Using VEGF-A as a proximal stimulus, Aicher et al. demonstrated that endothelial NO synthase becomes activated in BM stroma [38, 39]. By paracrine mechanisms, NO then S-nitrosylates and activates metalloproteinase-9 (MMP-9), which releases the stem cell active cytokine soluble kit ligand [37]. This agent shifts stem and endothelial progenitor cells from a quiescent to proliferative niche, stimulating rapid stem cell mobilization to the peripheral bloodstream [35, 38, 40].

### 2.3. Homing of Bone Marrow-Derived Endothelial Progenitor Cells to Site of Injury

Success of SCT relies on precise homing of the engrafted therapeutic and endogenous stem and progenitor cells to the target ischemic tissue, mediated by cell-cell interaction of infused exogenous and/or mobilized endogenous stem and progenitor cells with endothelial cells (ECs) in ischemic tissue vasculature. Hence, the ability to provide tissue regeneration and angiogenesis relies upon not only the potency of stem cells but also a hospitable ischemic tissue microenvironment that is receptive to either exogenous or endogenous stem and progenitor cells. Cell-cell interaction between circulating stem and progenitor cells and ECs in ischemic tissue vasculature is mediated by a panel of adhesion molecules, including E-selectin [41]. Elevated expression of E-selectin on the activated endothelium located within ischemic tissues is triggered by chemo-cytokines, such as SDF-1*α*, which mediates migration and homing of stem cells when induced by low oxygen sensor hypoxia-inducible factor-1*α* (HIF-1*α*) during tissue ischemia [41]. E-selectin highly expressed on activated endothelium serves as “docking sites” to anchor circulating EPCs and other stem cells which express counterpart E-selectin ligands. E-selectin ligand-mediated cell-cell interaction between circulating stem and progenitor cells and ECs in ischemic tissue vasculature is a fundamental event in stem and progenitor cell-induced tissue repair and angiogenesis [41, 42].

### 2.4. Therapeutic Angiogenesis

Blood vessels are critical for providing oxygen and nutrients, which are required for repair of injured tissue. Therapeutic angiogenesis promotes and enhances neovascularization in injured tissues through various approaches, including gene therapy or SCT, facilitating tissue repair. BM cells contain heterogeneous population of EPCs, MSCs, and hematopoietic stem cells that contribute to neovascularization to correct inadequate tissue perfusion [12]. For example, BM-derived EPCs given to animals with surgically induced limb ischemia incorporate into foci of neovascularization in ischemic muscle, skin, and wounds [43]. Neovascularization of the ischemic tissue occurs by the process of angiogenesis and vasculogenesis [37].

In normal quiescent vessels, ECs act as a multipurpose barrier between flowing blood and extravascular tissue. Periendothelial cells such as fibroblasts and smooth muscle cells reinforce these tubular networks and enhance endothelial cell-cell contact, produce extracellular matrix (ECM) proteins, and regulate the luminal diameter [44]. Initiation of angiogenesis requires destabilization of preexisting endothelial tubular structures by tissue injury followed by the release of cytokines and cell-matrix interactions that all play an instrumental role in activating ECs to begin angiogenesis [45]. Specifically, activated ECs, platelets, smooth muscle cells, monocytes/macrophages, and fibroblasts release the necessary proangiogenic cytokines such as VEGF-A, platelet-derived growth factor (PDGF), and transforming growth factor-beta (TGF-*β*), which allow local, resident ECs to invade and migrate through the ECM, proliferate, and form new immature tubules [45]. Not only do fibroblasts provide a rich source of proangiogenic cytokines and important stimulatory signals for angiogenesis but they also provide the ECM, which acts as the scaffolding for neovasculature [46]. As ECs migrate into the area of neovascularization, they further proliferate and form cytoplasmic vacuoles, which later become immature leaky tubules [45]. These tubules eventually become a functional barrier with the cooperation of the periendothelial cells, which enhance cell-cell junctions, lay down basement membrane, and secrete other ECM components [45].

**Figure 1:**
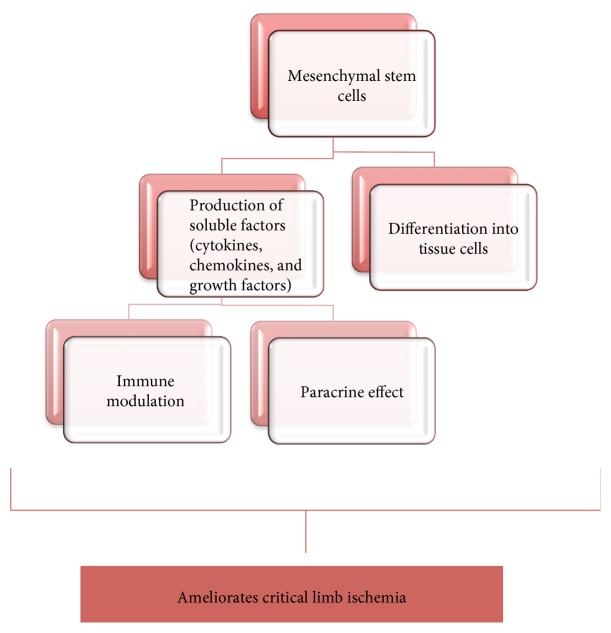
Potential mechanisms of mesenchymal stem cells in treating peripheral artery disease related critical limb ischemia.

On the other hand, vasculogenesis begins with multipotent adult progenitor cells, which differentiate into EPCs in the BM. Increased levels of VEGF-A enhance MMP-9 secretion, which leads to the secretion of soluble kit ligand. This ligand then mobilizes EPCs from the BM into circulation [45]. Once in circulation, early EPCs further differentiate to late EPCs and gain specific EC surface markers. Finally, late EPCs arrive to the site of neovessel formation and further differentiate into mature ECs for neovascularization [45]. Overall, SCT regulates therapeutic angiogenesis and tissue repair by diverse mechanisms, including (1) differentiation into ECs and/or various tissue cells to replace damaged cell types; (2) secretion of paracrine factors such as growth factors, cytokines, and hormones to regulate neovascularization; and (3) immune modulation and anti-inflammatory effects (Figure 1).

Ischemia itself is a stimulus for angiogenesis where ischemic conditioning (IC) is a method of angiogenic stimulus for limb ischemia. IC involves the application of a series of alternating intervals of brief ischemia and reperfusion in the setting of prolonged ischemia causing tissue necrosis. The conditioning stimulus can be applied before, during, or after the major ischemic event. All three methods of conditioning are associated with tissue protection not only in normal physiology but also in both animal models and in humans with ischemia-reperfusion syndromes. One study noted that rats exposed to IC after hindlimb surgery using the tourniquet method demonstrated significantly higher blood flow than those without IC [47]. Likewise, angiogenic scores up to 30 days after surgery for rats exposed to IC were significantly higher than those without IC [47]. The levels of EPCs in rats undergoing sham surgery were significantly lower than in those rats undergoing hindlimb surgery and IC [47]. Overall, studies have shown that IC in the critical ischemic limb is associated with better microcirculation, decreased leukocyte endothelial sticking and endothelial dysfunction, and better capillary blood flow with terminal arteriole dilation [47, 48].

The number and function of circulating BM-derived EPCs are severely impaired by certain risk factors such as diabetes, smoking, and advanced age, which lead to insufficient collateralization and are highly correlated with long-term cardiovascular and wound-healing sequelae [49]. An increasing number of clinical trials using BM-derived progenitor cells have demonstrated clinical benefit, showing improvement in objective and subjective measures of perfusion, pain reduction, increase in total walking distance, and most importantly, decreased rate of amputation [16, 17]. For all these reasons, cell-based therapy holds promise as potential novel therapeutic modality for patients with advanced PAD.

## 3. Preclinical Testing of Stem Cell Therapy

### 3.1. Efficacy of Stem Cell Therapy in Murine Hindlimb Ischemia Model

BM-derived stem and progenitor cells have been identified as a potential new therapeutic option to induce angiogenesis. Numerous studies have been explored in animal models where preclinical studies have established the safety, feasibility, and efficacy of stem cell-based therapy in CLI [50, 51]. In a murine model simulating PAD through surgical hindlimb ischemia, iPS cells generated from human fibroblasts were intramuscularly injected postoperatively with perfusion recovery measured by laser Doppler [52]. Significantly improved reperfusion and greater capillary density in the ischemic gastrocnemius muscle was demonstrated in treated mice as compared to control by postoperative day 14 [52]. Furthermore, on histology, there was significantly less myofiber heterogeneity, nuclear centralization, fatty degeneration, and fibrosis in iPS cell-treated hindlimbs as compared to controls, indicating less tissue damage [52, 53]. Similarly, transplantation of allogeneic adipose-derived regenerative cells has been shown to be a promising treatment modality for severe ischemic diseases [54]. Adipose-derived stem cells labeled with magnetically visible nanoparticles were injected into an old apolipoprotein E knockout mouse model with hindlimb ischemia, where *in vivo* tracking of labeled cells within the hindlimb was performed by magnetic resonance imaging (MRI) one month postoperatively [54]. MRI confirmed cell survival and engraftment, laser Doppler imaging demonstrated greater superficial blood flow in lower ischemic limbs after treatment, and histology revealed higher microvessel density in mice receiving adipose-derived SCT [54].

While the utility of SCT in hindlimb ischemia has been demonstrated, stem cells may exhibit varied differentiation potentials and thereby distinct clinical effects depending on the cellular source, despite having similar phenotypic and surface antigen expression [55]. MSCs are pluripotent cells derived from the BM that may differentiate into a variety of cells including endothelial and vascular smooth muscle cells. A comparison of angiogenic potency between BM-derived MSCs and mononuclear cells (MNCs) in a rat model of hindlimb ischemia was performed [50]. MSCs retained higher blood perfusion on laser Doppler imaging in addition to capillary density when compared to MNCs [50]. Furthermore, the number of transplanted cell-derived ECs was higher in the MSC group than in the MNC group, where the former was more tolerant to apoptotic stimuli such as serum starvation and hypoxia [50].

However, the clinical applicability of BM-derived EPCs and MSCs may be limited to the relatively invasive procedure required for sample collection as well as the marked reduction in cell number, proliferation, and differentiation capacity with age [55]. Therefore, multiple tissues have been investigated as alternatives to BM as a source of stem cells, including placenta, adipose tissue, fetal lung, dental pulp, and umbilical cord blood [55]. For example, intramuscular injected, placenta-derived MSCs have been shown to have a higher therapeutic efficacy than BM and adipose tissue-derived MSCs in murine hindlimb ischemia models [55]. Immunostaining studies suggest that injected placenta-derived MSCs can incorporate into the vasculature and differentiate into endothelial and smooth muscle cells to enhance angiogenesis in ischemic hindlimb [56]. These findings indicate that the choice of MSC source and purification protocol is critical in determining the therapeutic potential of these cells and warrants the standardization of an optimal MSC isolation procedure to select the best conditions for therapeutic angiogenesis.

The neovascularized capacity of EPCs derived from diabetic patients has been found to be impaired [57]. Therefore, investigation of the viability of allogenic healthy stem cells in the treatment of patients with both diabetes and ischemia is urgently needed.

In one study, diabetic nude rats were randomly divided into several groups as follows: diabetic ischemic nude rats transplanted with MSCs at 2 × 10^6^ (high-dose group) or 0.5 × 10^6^ (low-dose group); diabetic ischemic nude rats treated with insulin alone; the combination therapy groups of diabetic ischemic nude rats treated with insulin and MSCs at high dose or low dose; and diabetic ischemic nude rats transplanted with vehicle (PBS control group) [58]. MSCs improved ischemia damage and functional recovery in diabetic rats; however, the combination therapy of cell treatment and insulin injection did not show increased improvement [58]. The recovery of ischemic damage was significantly improved by cell therapy at high dose at days 17 and 28, but not by insulin injection alone or by combination therapy [58]. Furthermore, improvement in ischemic damage was similar between high-dose and low-dose MSC groups [58].

Three weeks later, blood flow perfusion was restored to some degree in all groups, but did not return to normal in the PBS group where the ischemia/nonischemia perfusion ratio was only 57% [58]. The perfusion recovery in the cell transplant groups was significantly higher (89% in the high-dose group versus PBS, *P* < 0.01; 83% in the low-dose group versus PBS, *P* < 0.01) [58]. These results reveal that MSCs improve blood flow perfusion and that the higher dose of cells is more effective in doing so [58]. High-dose MSCs showed significant improvement in the restoration of blood flow when compared to PBS group [58]. MSCs accelerate collateral vessel formation, and while insulin has a positive effect on angiogenesis, it did not cooperate with the therapeutic effect of MSCs [58]. Therefore, this study indicates that cell therapy may be a promising new approach for diabetic CLI.

## 4. Clinical Testing of Stem Cell Therapy

### 4.1. Efficacy of Stem Cell Therapy in Patients with Critical Limb Ischemia

Patients suffering from lower extremity nonhealing ulcers or gangrene caused by PAD-induced CLI or diabetes are at high risk for major amputation and experience overall poor physical function with severely diminished quality of life. Despite the progress in medical and surgical therapy of patients with CLI, the prognosis of patients with no option for revascularization remains poor; the amputation rate is high with mortality rate approaching 20% within six months [1]. Novel therapeutic strategies involving SCT have been proposed during the last two decades in an effort to induce therapeutic angiogenesis and promote tissue regeneration in no-option patients. Therapies involving stem cell implantation have been tried in human subjects using defined experimental outcomes such as pain relief, walking distance, and wound healing in addition to limb salvage or amputation-free survival.

The encouraging results of aforementioned preclinical studies have rapidly led to several small clinical trials [14, 18, 59, 60]. A landmark, first-in-man clinical trial reported by Tateishi-Yuyama et al. showed safety and promising effects of autologous BM-derived cell therapy in patients with CLI [18]. In 1997, Asahara et al. and Shi et al. identified a class of BM-derived circulating EPCs that contribute to angiogenesis and/or vasculogenesis in ischemic tissues and improved tissue perfusion [61, 62]. Since then, research focusing on the use of autologous cell therapy has shown promising results and has led to several larger clinical studies in the last ten years, wherein autologous BM cell implantation in patients with CLI who had no other alternative treatment option showed safety and efficacy based on statistical analysis. These studies have also reported the capacity of BM stem and progenitor cells to promote revascularization, thereby improving limb perfusion sufficiently to resolve pain at rest, resulting in limb salvage.

The CLI-stem cell therapy (CLI-STEM) nonrandomized, single-center study evaluated the safety and therapeutic effectiveness of autologous BM cells in revascularization of CLI patients utilizing a rapid point of care device entitled Res-Q 60 BMC, an automated cell processing medical device that concentrates the BM by a density gradient centrifugation method [12]. Single dose of autologous BM cell concentrate was injected intramuscularly into the afflicted ischemic limb of 17 patients who met study inclusion criteria, revealing 82% amputation-free survival rate and a significant improvement in ankle brachial index (ABI), transcutaneous oxygen pressure (TcPO_2_), mean rest pain and intermittent claudication pain scores, wound/ulcer healing, and six-minute walking distance following autologous BM cell concentrate treatment [12]. Overall, this study demonstrated the safety and preliminary effectiveness of harvesting and injecting intramuscularly autologous BM cell concentrate in patients with no-option CLI, laying the platform for more pivotal trials.

While recent evidence indicates that BM cells promote collateral vessel formation in patients with severe PAD, aspects concerning optimal administration mode require consideration. One study evaluated the safety and effect of exclusive intramuscular (*n* = 15) versus combined intra-arterial plus intramuscular BM cell delivery (*n* = 12) in patients who were not candidates for surgical or endovascular treatment [63]. Two patients in the combined group required limb amputation because of ongoing CLI versus 7 patients in the intramuscular group (*P* = 0.17) [63]. BM cell treatment in the remaining patients resulted in a significant and sustained improvement (>12 months) with mean ABI increased 23% after 6 months (*P* = 0.01) and pain score reduced for up to 50% as shown by the Brief Pain Inventory (*P* = 0.001) [63]. The authors concluded that both intramuscular and combined therapy with BM cells are safe and result in relevant and sustained improvement in a considerable proportion of patients with severe PAD [63]. While this study showed no significant difference in terms of these two strategies, it was underpowered to detect differences in terms of ulcer healing, ABI, and walking distance improvement. Upon meta-analysis, ABI and TcPO_2_ were significantly improved after intramuscular cell therapy, while they were not after intra-arterial cell therapy [64]. Both significantly improved pain and pain-free walking distance, but there was no difference between the two [64].

Currently, several large randomized, placebo-controlled, double-blind studies, including RESTORE-CLI, BONMOT-CLI, JUVENTUS, NCT00498069, NCT01049919, NCT01245335, and NCT00919958, are underway [13–15, 65, 66]. RESTORE-CLI is a prospective, randomized double-blinded and placebo-controlled multicenter study conducted at 18 centers in the United States in patients with CLI and no option for revascularization [13]. The interim results from RESTORE-CLI are very promising and show that intramuscular injection of autologous BM-derived TRCs including stem and progenitor cells is safe and well tolerated with no significant difference in adverse events between groups [13]. TRCs decreased the occurrence of clinical events associated with disease progression when compared to placebo in patients with no-option CLI [13]. Specifically, treatment with TRCs improved time to treatment failure, defined as major amputation, all-cause mortality, doubling of total wound surface area from baseline, or de novo gangrene, in addition to improvement in amputation-free survival [13]. These results clearly suggest that TRCs have the potential to be a promising treatment option in patients with CLI who are not eligible for revascularization, representing an important advance in research related to regenerative medicine [13].

While the majority of trials have tested a potential effect of intramuscular injection, the PROVASA trial is a multicenter phase II trial with a double-blind randomized-start design that tested intra-arterial treatment with BM-derived MNCs in 40 patients with CLI [59]. While intra-arterial administration of BM-derived MNCs did not significantly increase ABI, missing the primary endpoint of the trial, cell therapy was associated with significantly improved ulcer healing and reduced rest pain versus placebo within three months [59]. Despite improvements in secondary endpoints, critically ill patients with impending amputation did not derive any benefit from BM MNC administration given that amputation-free survival rates did not differ between groups [59]. Nonetheless, the proven procedural safety profile of BM harvest and intra-arterial BM MNC administration provides the necessary framework for a repeated treatment strategy in larger clinical trials that can be easily implemented into clinical guidance of patients with CLI.

At present, many phase III clinical trials are either actively enrolling or are in early stages of development. Pluristem Therapeutics is a leading developer of placenta-based stem cell therapy products [26]. Their Phase III trial will evaluate placenta expanded- (PLX-) PAD cells in the treatment of CLI in a double-blind, multinational, randomized, placebo-controlled trial. An estimated 250 patients with CLI Rutherford Category 5, who are unsuitable candidates for revascularization, will be enrolled. Patients will be treated with 300 million cells or placebo, injected twice intramuscularly two months apart. The primary endpoint will be time to amputation or death. SCT, if proven effective in phase III trials, might become a useful adjunct to the current treatment options for no-option patients with CLI.  Table 1 summarizes completed and ongoing clinical trials of SCT in patients with PAD/CLI.

Current literature underscores certain factors associated with therapeutic benefits after autologous BM cell therapy in patients with “no-option” CLI. In a study with 62 patients diagnosed with advanced CLI and randomized to treatment with autologous BM cells by local intramuscular or intra-arterial application, the primary endpoint was limb salvage and wound healing at 12 months [67]. The BM cell product of patients with limb salvage and wound healing (33/55) was characterized by a higher CD34^+^ cell count (*P* = 0.001), as well as higher number of total BM MNCs (*P* = 0.032) than that of nonresponders (22/55) [67]. Patients with limb salvage and wound healing were younger (*P* = 0.028), had lower C-reactive protein levels (*P* = 0.038), and had higher TcPO_2_ (*P* = 0.003) before cell application than nonresponders [67]. Furthermore, all patients with major tissue loss at baseline showed the progression of limb ischemia and required major limb amputation [67]. Multiple logistic regression showed that the number of applied CD34^+^ cells (*P* = 0.046) and baseline TcPO_2_ (*P* = 0.031) were independent predictors of limb salvage and wound healing [67]. The number of administrated BM MNCs strongly correlated with decreased peripheral leukocyte count after 6 months in surviving patients with limb salvage (*P* = 0.0008) [67]. These data indicate that age in addition to higher doses of CD34^+^ and BM MNCs may correlate with higher amputation-free survival rates, thereby differentiating responders from nonresponders.

### 4.2. Challenges and Limitations of Stem Cell Therapy

In spite of very promising results from numerous clinical trials, many open questions and challenges remain in regard to SCT. In addition to understanding the precise molecular mechanisms underpinning the beneficial effects of stem and progenitor cell therapy, we have much to learn about this new treatment modality. First, we need to identify the most ideal cell type—for example, unfractionated BM MNCs, peripheral blood MNCs, BM-derived EPCs, BM-derived MSCs, adipose-derived stem cells, and iPS cells, which may be suitable for cell-based treatment. Secondly, a better understanding of the effective subpopulation of stem cells is necessary as stem cells are heterogeneous population. Not all subpopulations are equally effective. Improvements to cell therapy will benefit from a more precise characterization of cellular subsets in the therapeutic product. Thirdly, we must develop effective large-scale *ex vivo*/*in vitro* cell differentiation protocols or cell isolation methods if a subpopulation of a specific cell lineage turns out to work better in preclinical small animal models. Fourthly, it will be important to establish efficient methods for enhancement of cell potency before administration (*ex vivo* stimulation/treatment of stem cells with cytokines and growth factors, for example, SDF-1*α*, G-CSF, hepatocyte growth factor, and fibroblast growth factor). Lastly, it remains to be determined what is the optimal dosage of therapeutic cells, which one is the best route of administration (intramuscular versus intra-arterial versus systemic targeted delivery) and what is the ideal frequency of application. Ultimately, we will also need to study the impact of autologous versus allogeneic stem cell implantations and understand the tissue endogenous microenvironmental factors, such as hypoxia, oxidative stress, and diabetes mellitus, that affect the therapeutic activity or in situ differentiation/maturation capability of the applied stem and progenitor cells.

PAD/CLI is associated with both microvascular and macrovascular abnormalities. Whereas increased microvascular density is one of the beneficial outcomes of SCT-mediated therapeutic angiogenesis, which helps to improve perfusion to ischemic tissue, the ischemic human leg requires larger conduits, such as collateral artery formation that provides sufficient inflow. Clearly, new insights into the mechanisms of SCT-induced arteriogenesis are also needed so as to develop effective methods to therapeutically manipulate arteriogenesis.

An alternative to SCT may be EPC-derived microvesicles (MV), which are able to activate an angiogenic program in quiescent endothelial cells by a horizontal transfer of RNA. Ranghino et al. investigated whether EPC-derived MVs are able to induce neoangiogenesis and to enhance recovery in a murine model of hindlimb ischemia [68]. Hindlimb ischemia was induced in severe combined immunodeficient (SCID) mice by ligation and resection of the left femoral artery, and mice were treated with EPC-derived MVs. The limb perfusion evaluated by laser Doppler analysis demonstrated that MVs significantly enhanced perfusion in respect to control [68]. After 7 days, immunohistochemical analyses on the gastrocnemius muscle of the ischemic hindlimb showed that MVs significantly increased capillary density in respect to control [68]. The results of the present study indicate that treatment with EPC-derived MVs improves neovascularization and favors regeneration in severe hindlimb ischemia-induced SCID mice. This suggests a possible use of EPC-derived MVs for the treatment of PAD.

### 4.3. Concluding Remarks on Stem Cell Therapy

As shown in this review, numerous clinical trials have shown promising results and demonstrated the beneficial role of SCT in reducing the rate of major amputation, improving distal perfusion, increasing walking distance, reducing pain, improving ABI and TcPO_2_, and improving overall ischemic symptoms in patients with CLI and their quality of life. In addition, the procedures of SCT are generally safe and well tolerated. Compelling evidence of clinical safety and efficacy provides solid rationale for ongoing in depth studies aimed at developing novel SCT that could dramatically alter how we care for patients with PAD/CLI and recalcitrant lower extremity diabetic wounds.

## Figures and Tables

**Table 1:** Clinical trials using stem cells for treatment of peripheral artery disease related critical limb ischemia.

Trial number	Phase	Study period	Route	Treatment
NCT00883870	1 and 2	4/2009–3/2011	IM	*Ex vivo* cultured adult allogeneic MSCs versus plasmalyte A
NCT00616980	1 and 2	12/2007–10/2010	IM	Autologous CD34^+^ cells
NCT00919958	1	06/2009–06/2010	IM	Allogeneic PLX-PAD
NCT00951210	1	08/2009–10/2011	IM	Allogeneic PLX-PAD
NCT01049919	1 and 2	06/2010–05/2014	IM	Autologous concentrated BM aspirate using MarroStim PAD kit
NCT00468000	2	04/2007–03/2011	IM	Autologous BM cells
NCT00987363	1 and 2	07/2009–12/2011	IA	Autologous BM-derived MNCs
NCT01019681	1	11/2009–11/2015	IM	Umbilical cord blood stem cells
NCT00872326	1 and 2	12/2007–05/2009	IA	Autologous BM-derived MNCs
NCT00523731	1	01/2006–03/2007	IM	Autologous, nonmobilized angiogenic cell precursor
NCT00392509	1 and 2	10/2006–12/2008	IM	Aldehyde dehydrogenase bright stem and progenitor cells
NCT01079403	1 and 2	12/2009–12/2011	IA	Autologous adipose tissue-derived MSCs
NCT00498069	1 and 2	11/2007–12/2014	IM	Autologous BM aspirate
NCT00922389	1 and 2	07/2009–01/2011	IM	G-CSF^+^ PB-derived MNCs
NCT00913900	1	05/2009–09/2012	IM	Autologous CD133^+^ cells
NCT00371371	1 and 2	11/2006–07/2013	IA	Autologous BM-derived MNCs
NCT00721006	2	11/2006–12/2010	SC (40x)	Combination of SCT
NCT01065337	2	10/2004–02/2009	IM/IA	Autologous BM-derived MNCs versus tissue repair cell CD90^+^ cells
NCT00533104	1 and 2	10/2004–02/2009	IM (30x)	Autologous PB-derived MNCs and BM-derived MNCs
NCT00595257	1 and 2	12/2007–08/2010	IM	Autologous BM aspirate using SmartPREP2
NCT00434616	2 and 3	04/2007–07/2011	IM	Autologous BM cell concentrate
NCT00904501	3	03/2009–06/2014	IM	Autologous BM-derived MNCs
NCT00488020	1	04/2006–06/2007	IM (40x)	Autologous BM-derived MNCs
NCT00518401	1	06/2007–10/2009	IM (40x)	Combination of stem cell mixture
NCT00221143	1 and 2	11/2003–01/2008	IM (40x)	Autologous PB CD34^+^ cells
NCT00539266	2 and 3	10/2007–10/2010	IM	Autologous BM-derived MNCs (DM versus non-DM)
NCT00145262	2	08/2003–	IM	Autologous BM-derived MNCs
NCT00282646	1 and 2	10/2005–03/2011	IA	Autologous BM-derived MNCs
NCT02538978	3	08/2015–07/2016	IM	Autologous BM cells using SurgWerksTM-CLI kit and VXPTM system
NCT01386216	1	06/2011–01/2017	IM	Autologous BM aspirate using Magellan system
NCT01558908	1 and 2	03/2012–	IM	Endometrial regenerative cells
NCT02551679	2	11/2015–	IM	Autologous angiogenic cell precursors
NCT02140931	2	05/2014–11/2015	IM	Autologous angiogenic cell precursors

BM: bone marrow; BM-MNC: bone marrow mononuclear cell; DM: diabetes mellitus; G-CSF: granulocyte colony-stimulating factor; IA: intra-arterial; IM: intramuscular; MSC: mesenchymal stem cell; PAD: peripheral arterial disease; PB: peripheral blood; PB-MNC: peripheral blood mononuclear cell; PLX-PAD: placental-derived mesenchymal stem cells; SC: subcutaneous.
